# Effectiveness of pocket ark 2.0 in enhancing post-flood response and improved knowledge of workers: a pilot test study

**DOI:** 10.3389/fpubh.2026.1831287

**Published:** 2026-08-27

**Authors:** William Brett Perkison, Dejian Lai, Ross Shegog, Geethika Yalavarthy, Hye Seung Lucy Lee, Rosalia Guerrero-Luera, Jefferey McLaughlin, Ismail Nabeel

**Affiliations:** 1 Department of Environmental Health Sciences, University of Texas School of Public Health, Houston, TX, United States; 2 Department of Health Promotion, University of Texas School of Public Health, Houston, TX, United States; 3 The Institute of Aging, The University of Texas School of Public Health, Houston, TX, United States; 4 Department of Management, Policy, and Community Health, University of Texas School of Public Health, Houston, TX, United States; 5 New Math Data, Houston, TX, United States; 6 Department of Environmental Medicine and Public Health, Icahn School of Medicine, New York, NY, United States

**Keywords:** disaster recovery, e-learning, occupational health, pocket ark, post-flood, public health preparedness, reconstruction workers, technology acceptance

## Abstract

Flood recovery workers, also known as reconstruction workers, face elevated occupational risks, including exposure to hazardous environmental conditions, infectious agents, and unsafe labor practices, yet often lack access to timely, culturally appropriate safety training and advocacy resources. *Pocket Ark 2.0, a mobile application software,* was developed as a bilingual, mobile digital decision-support and training platform designed to enhance occupational safety, preparedness, and worker advocacy during post-flood disaster recovery. The objective of this pilot study was to evaluate the usability, technology acceptance, and knowledge outcomes associated with *Pocket Ark 2.0* among reconstruction workers participating in a simulated disaster-response training. Using a single-group, post-test study design, 55 reconstruction workers affiliated with a community-based worker advocacy organization completed interactive, scenario-based modules covering personal protective equipment, wage documentation, hazard reporting, communication, and resource navigation. Outcomes included pre- and post-knowledge assessments, the System Usability Scale (SUS), and the Technology Acceptance Model (TAM). Participants demonstrated improvements in occupational safety knowledge following the simulation. Usability outcomes were strong, with a median SUS score of 86.25, indicating excellent perceived usability. Technology acceptance was high, with most participants reporting favorable perceptions of usefulness and ease of use. Language accessibility and prior familiarity with digital tools were significant predictors of usability and acceptance, underscoring the importance of culturally and linguistically tailored design. These findings support the feasibility of *Pocket Ark 2.0* as a scalable, community-embedded digital intervention for enhancing safety training, preparedness, and advocacy among disaster recovery workers. Digital decision-support platforms that integrate education, communication, and resource coordination may represent an effective strategy for advancing occupational health equity in disaster response settings.

## 1 Introduction

### 1.1 Background

Flooding events continue to increase globally in both frequency and intensity, with reports of natural disasters tripling between 1960 and 2007 (1, 2). Workers involved in post-flood clean-up and reconstruction remain particularly vulnerable, facing risks such as respiratory and skin exposure to irritants, waterborne infectious agents, electrocution, and mold-related illnesses (3, 4). These risks are compounded by inadequate access to personal protective equipment (PPE) and susceptibility to wage exploitation (3). Despite updated federal guidance, under-use of respirators, gloves, and waterproof boots continues to be reported (4). Occupational Safety and Health Administration (OSHA) has repeatedly advised that insufficient PPE leaves recovery workers vulnerable to infection, chemical exposure, and injury (5). National analyses confirm that wage theft remains disproportionately concentrated in disaster-recovery contexts (3). Protecting this essential yet underserved workforce has therefore become an urgent public-health priority in light of recent natural disaster events.

*Pocket Ark 1.0* was designed as a mobile e-learning and decision-support tool to meet the safety, health, and advocacy needs of reconstruction workers (6). Designed in partnership with Radiant Digital Inc., (Vienna, VA, USA), the platform provides scenario-based PPE training, wage-negotiation support, resource location, and healthcare guidance tailored to the demographic, cultural, and educational characteristics of this workforce. It also incorporates bidirectional communication between workers and supervising coordinators to facilitate safety logistics (6). The theoretical foundation for the development of *Pocket Ark 1.0* draws on social behavior theory (7, 8) and the Conceptual Framework for Organizational Innovation Adoption (CFOIA) (9). Integrating these frameworks provided a dual focus on individual learning outcomes and organizational readiness for technology integration. By embedding interactive modules that simulate real-world scenarios, the mobile application software delivers safety information while reinforcing self-efficacy and collective norms: key elements of sustained behavioral change (6, 10).

*Pocket Ark 1.0* has undergone multiple iterative pilots. We collaborated with Resilience Force, a New Orleans–based worker-advocacy organization supporting reconstruction workers who restore disaster-affected communities (11). The organization provides workforce support, safety training, and advocacy services to a regional network of approximately 100 workers. This population represents a community of frontline reconstruction workers with direct experience in post-disaster environments, making it well-suited for evaluating the feasibility and usability of the intervention. Earlier pilots demonstrated the feasibility of *Pocket Ark 1.0* in improving PPE knowledge and usability (6). Subsequent iterations refined interface usability, expanded bilingual support, and strengthened modules related to wage negotiation and communication (6). Based on these findings, we developed *Pocket Ark 2.0*, a fully integrated bilingual version with offline functionality and Geographic Information System (GIS)-enabled mapping (6).

### 1.2 Previous work

*Pocket Ark 1.0* has been iteratively developed and tested over multiple pilots (6). We collaborated with Resilience Force, a worker advocacy organization based in New Orleans, Louisiana, USA dedicated to assisting construction workers who specialize in restoring infrastructure in communities devastated by natural disasters (termed reconstruction workers) (6, 11). In each of our previous pilots we have recruited reconstruction workers who have been affiliated with Resilience Force’s extensive network of contacts. In *Pocket Ark 1.0*, a user-informed prototype incorporated PPE training, hazard recognition, and communication functions. When COVID-19 emerged, the platform was adapted to provide pandemic-specific safety information, demonstrating its flexibility. The feasibility testing of this pilot study showed significant knowledge gains and favorable usability ratings, with workers reporting high confidence in its utility (6).

The subsequent pilots, which are described in a previous publication (6, 11), refined the application based on feedback to improve interface usability, enhance multilingual support, and strengthen modules related to wage negotiation and communication. Informal debriefings and survey data showed high acceptance: 67% strongly agreed Pocket Ark (PA) was easy to use, 83% agreed its functions were well integrated, and 75% agreed it would improve their job performance (6).

Based on these findings, we developed *Pocket Ark 2.0*, a fully integrated bilingual version supporting offline access, GIS-enabled mapping, and automated data capture, representing the final prototype evaluated in the present study (6, 11). Internal testing with University of Texas Health Science (UTHealth Houston) staff and Resilience Force advocates confirmed technical stability and usability across devices. These refinements positioned *Pocket Ark 2.0* for full-scale simulation testing to assess usability, technology acceptance, and knowledge gains among reconstruction workers during realistic disaster-response scenarios. Accordingly, the objective of the present study was to evaluate these outcomes and determine the feasibility of *Pocket Ark 2.0* as a scalable intervention to enhance occupational safety, preparedness, and advocacy within the disaster-recovery workforce.

## 2 Methods

### 2.1 Study design and setting

The study utilized a single-group, post-test design to evaluate the usability and feasibility of *Pocket Ark 2.0.* We collaborated with Resilience Force to recruit study participants from among local members in their network. The study was conducted at their headquarters in New Orleans, Louisiana, USA and the data corresponded to an existing worker training session that had already been scheduled. Resilience Force holds regular in-person training for workers covering various health and safety topics. The study is approved by Center for the Protection of Human Subjects at UTHealth School of Public Health (HSC-SPH-23-0741).

### 2.2 Recruitment

Outreach was conducted through multiple strategies, including social media and WhatsApp messaging (12), to inform members of the Resilience Force network about the training sessions and study opportunity. Interested individuals were screened for eligibility and scheduled by Resilience Force staff to attend one of two in-person training sessions.

Formal recruitment and enrollment into the study occurred at the training site. Inclusion criteria included: age 18–65 years, affiliation with Resilience Force, prior construction experience, and access to an internet-capable smartphone (≤3 years old). Exclusion criteria included inability to commit to completing the questionnaires.

### 2.3 Study participants

**Figure 1:**
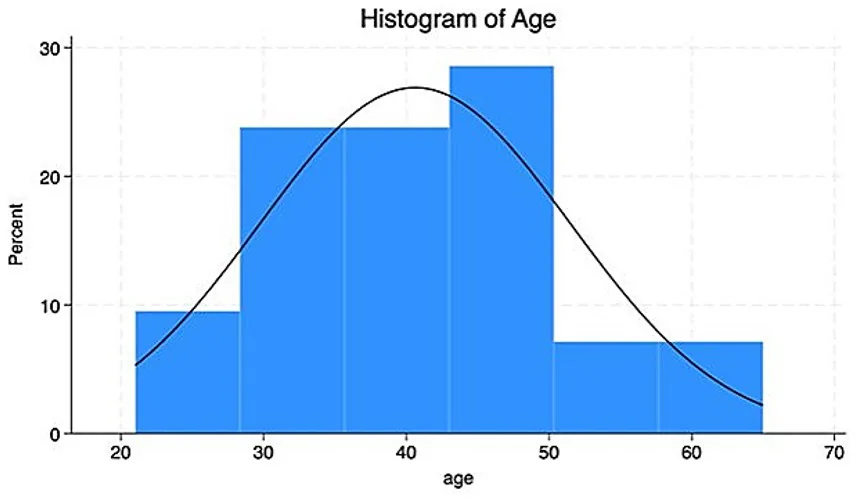
Histogram of participant age.

The total pool of potential participants was estimated at approximately 100 workers within the Resilience Force regional network during the study period. Participation was voluntary, and incentives were provided by Resilience Force in the form of lunch and health and safety supplies. Age of the participants enrolled ranged from 21 years old to 65 years old, with the mean age being 40.62 years old (See Figure 1). Of these, a total of 64 workers were recruited 32 per training day. Of these, 55 participants completed the study in full. Among participants, 39 (71%) preferred Spanish as their primary phone language, 6 (11%) preferred English, 6 (11%) reported no preference, and 4 (7%) did not provide a response.

9 participants were excluded from final analysis due to incomplete survey responses (*n* = 5), technical limitations with older smartphones (*n* = 2), or voluntary withdrawal prior to the post-survey (*n* = 2). These excluded cases were retained for descriptive reporting but excluded from outcome analyses.

The excluded participants ages ranged from 28 to 54 years. 5 (56%) reported no preference for a specific phone language, while 4 (44%) preferred Spanish. Educational attainment varied widely, ranging from elementary-level education to college graduates and widely varied computer literacy as well. Statistical analyses were conducted using SAS software.

### 2.4 Study procedure

Preparation involved coordination between the UTHealth Houston and Resilience Force teams to configure both classroom and outdoor learning stations used in the simulation. Coupling the *Pocket Ark 2.0* session with Resilience Force’s routine safety-training program allowed participants to engage within an authentic educational context while minimizing potential sampling bias arising from recruitment limited to app evaluation. Spanish- and English-speaking facilitators were present throughout to ensure comprehension and comfort among all participants.

Descriptive statistics were calculated for participants’ characteristics. Paired t-tests were used to assess differences in pre- and post-knowledge scores, and logistic regression analyses were performed to examine factors associated with outcomes.

**Figure 2:**
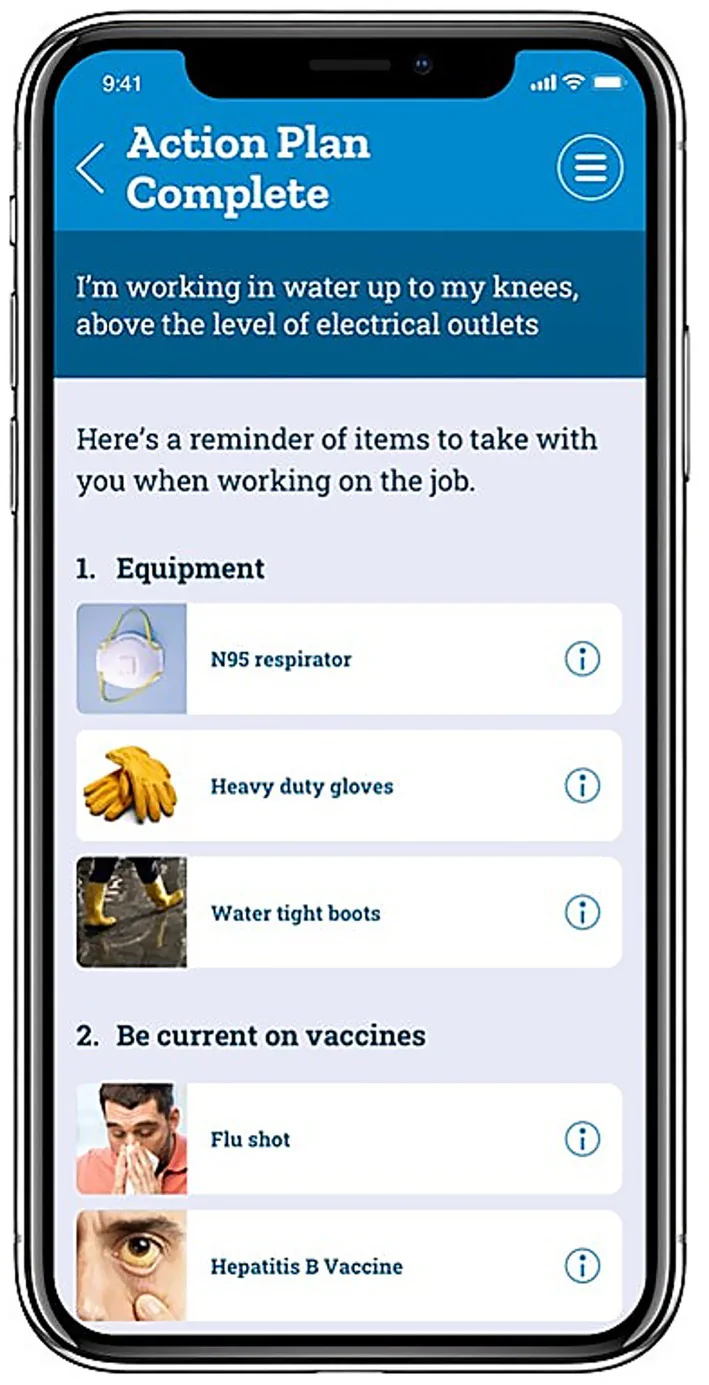
Workplace safety/PPE module.

**Figure 3:**
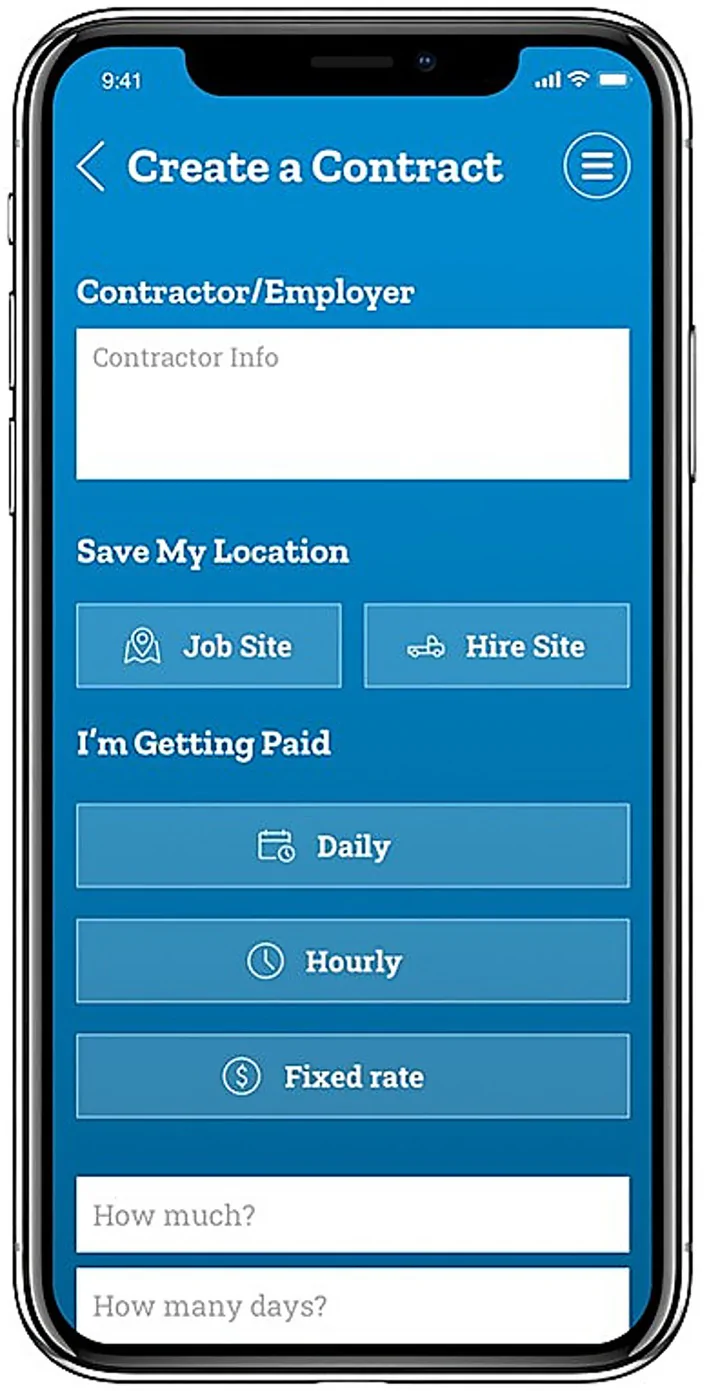
Wage negotiation module.

**Figure 4:**
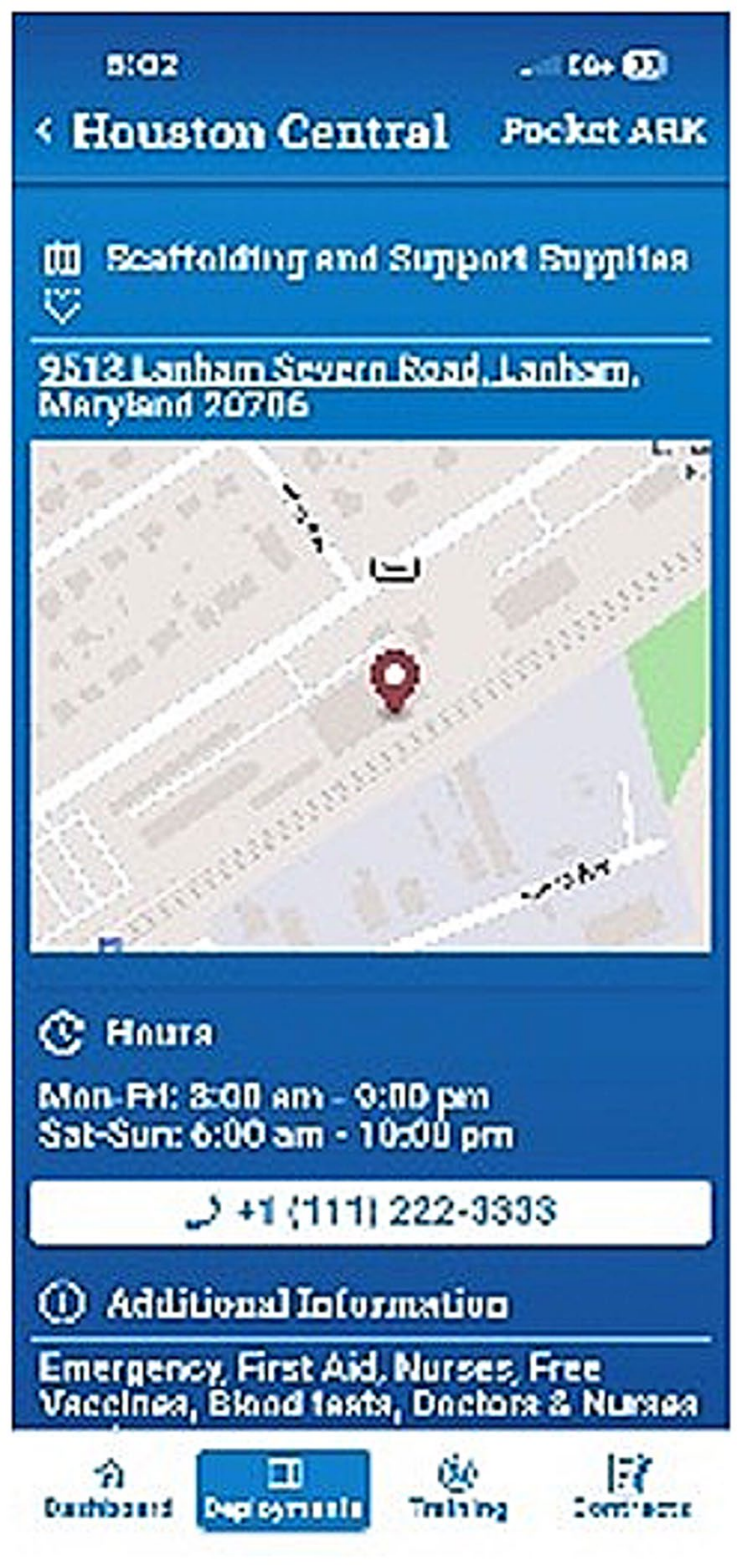
Resource locator module.

**Figure 5:**
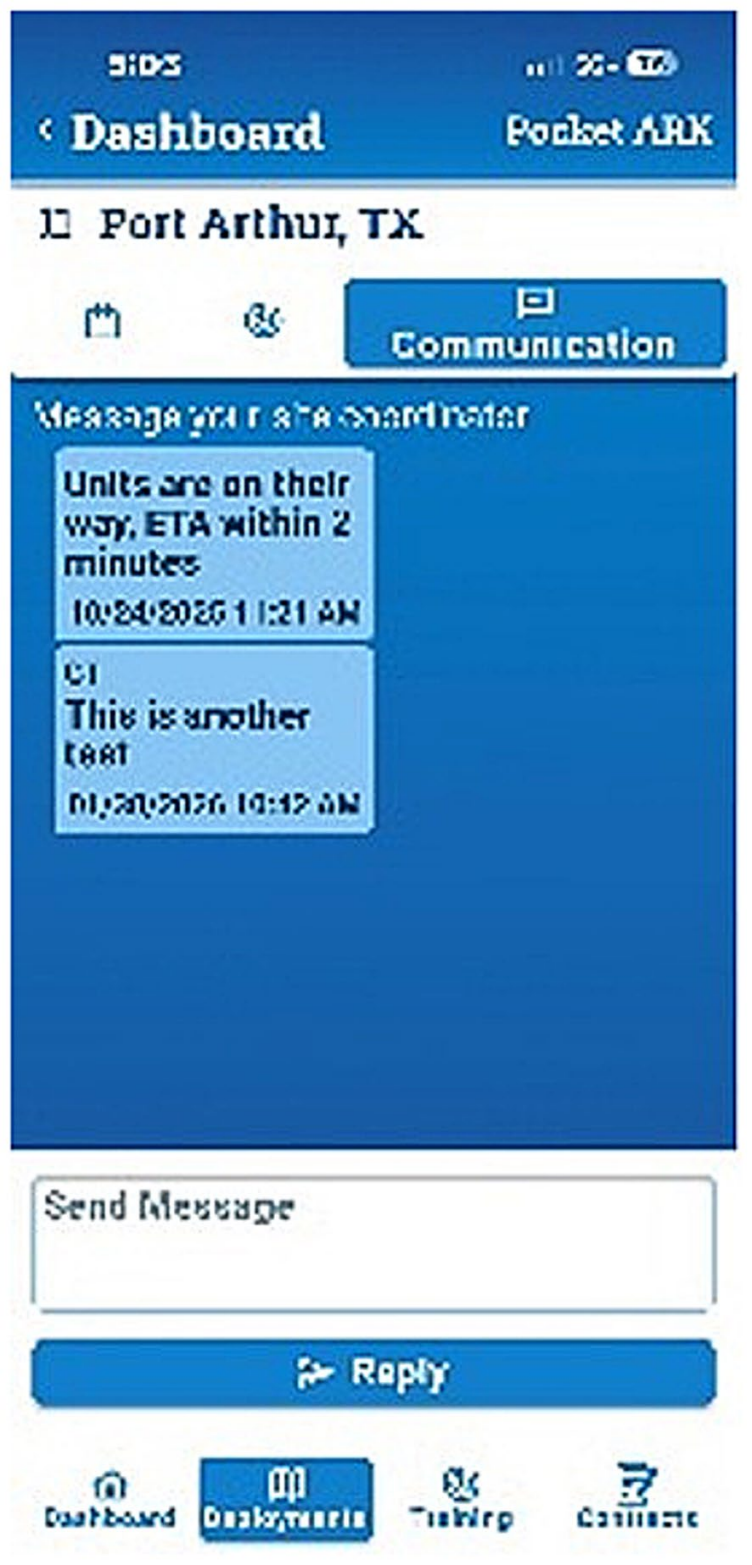
Communication module.

Each session followed a standardized structure:

1. Orientation: presentation describing *Pocket Ark 2.0*’s purpose, features, and study procedures (Spanish with English support).
2. Consent and pre-survey: participants received letters of information, unique study IDs, and completed pre-surveys (information includes demographics, digital familiarity, prior training, 10 knowledge questions including PPE knowledge, safety practices, hazard recognition and information regarding how the participant’s wages were negotiated, i.e., worker rights).
3. App access: participants scanned a QR code to access the clickable *Pocket Ark 2.0* prototype.
4. Interactive modules.

- Workplace safety/PPE module – identification of appropriate PPE for post-flood environments (Figure 2).
- Wage negotiation module – allows the worker to document a wage negotiated with the employer, type of job, as well as start and end dates (Figure 3).
- Sample job site module – digital job-site enrollment and submission of location specific data.
- Resource locator module – navigation to nearby food, lodging, supplies, and clinics (Figure 4).
- Communication module – exchange of discovered resources and hazard information with advocacy staff (Figure 5).

### 2.5 Measurements

The participants’ ability to use the app was measured using the following tools:

1. Pre/post knowledge survey – participants were tested on 10 questions regarding safety and PPE knowledge.
2. System usability scale (SUS) questionnaires were used to understand usability and acceptance. SUS is a 10-item questionnaire scored from 0 to 100, where scores above 70 indicate good usability, and scores above 80 indicate excellent usability (13).
3. Technology Acceptance Model (TAM) is a 12-item scale scored from 1 to 7, where scores greater than 6 reflect high acceptance in terms of perceived usefulness and ease of use. Spanish translation was provided as needed (14).

### 2.6 Analysis

Paired t-tests were used to assess differences in pre- and post-knowledge scores, and logistic regression analyses were performed to examine factors associated with outcomes.

## 3 Results

### 3.1 Knowledge test

The mean pre-test score was 7.26 (SD 1.09), while the mean post-test score was 7.67 (SD 1.24), reflecting a mean increase of 0.41 points. A greater proportion of participants achieved scores of 8 or 9 in the post-test compared to the pre-test.

### 3.2 System usability scale (SUS)

**Figure 6:**
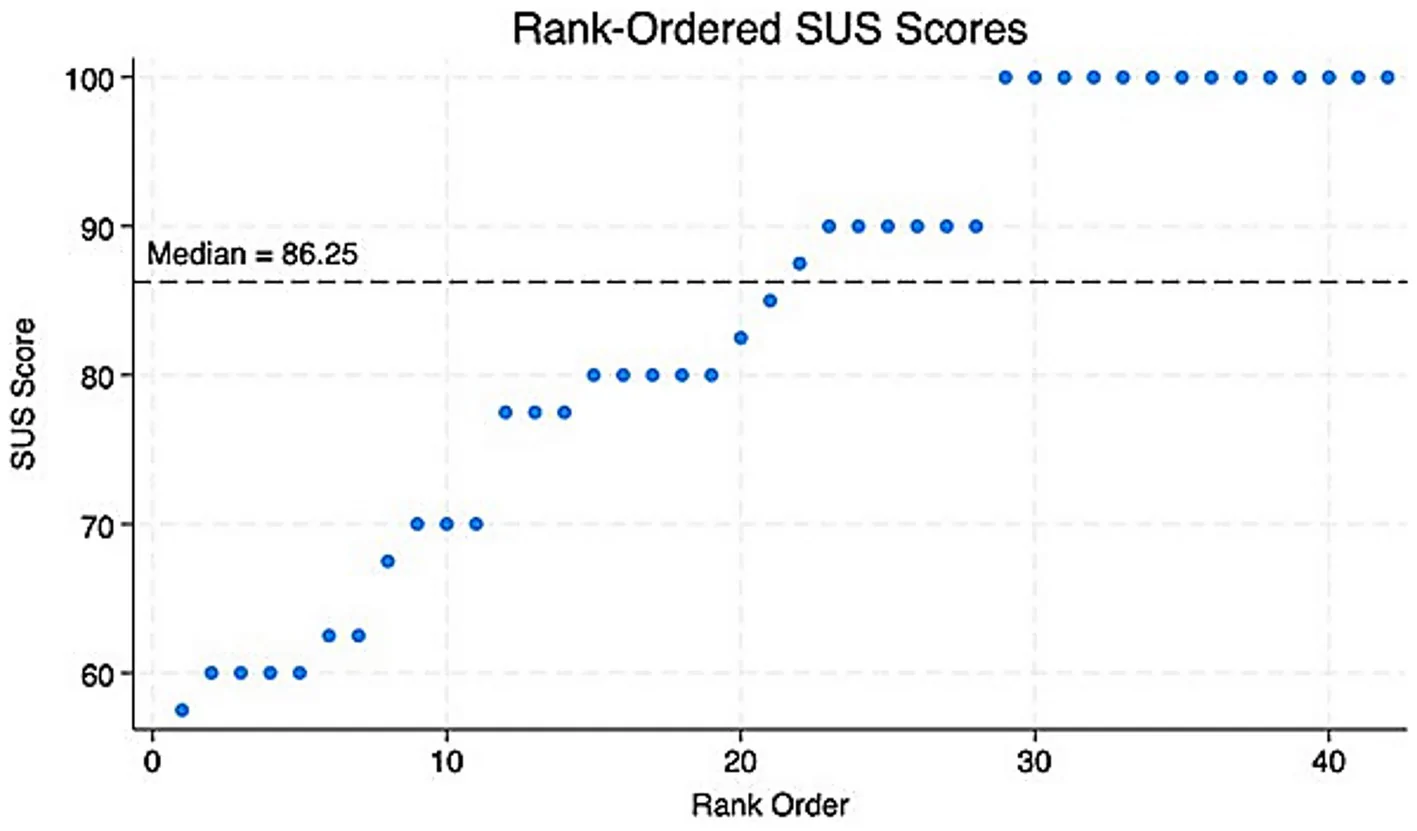
Scatterplot of rank-ordered system usability score (SUS).

The median SUS score was 86.25 and the mode was 100 (Figure 6). Approximately 80.9% of respondents scored above 70, the accepted threshold for “good” usability. Regression modeling revealed that language preference for phone operation was significantly associated with SUS scores (*p* = 0.0287), suggesting that users operating their devices primarily in Spanish reported higher perceived usability. No significant associations were observed for age, education, or prior construction experience.

### 3.3 Technology acceptance model (TAM)

**Figure 7:**
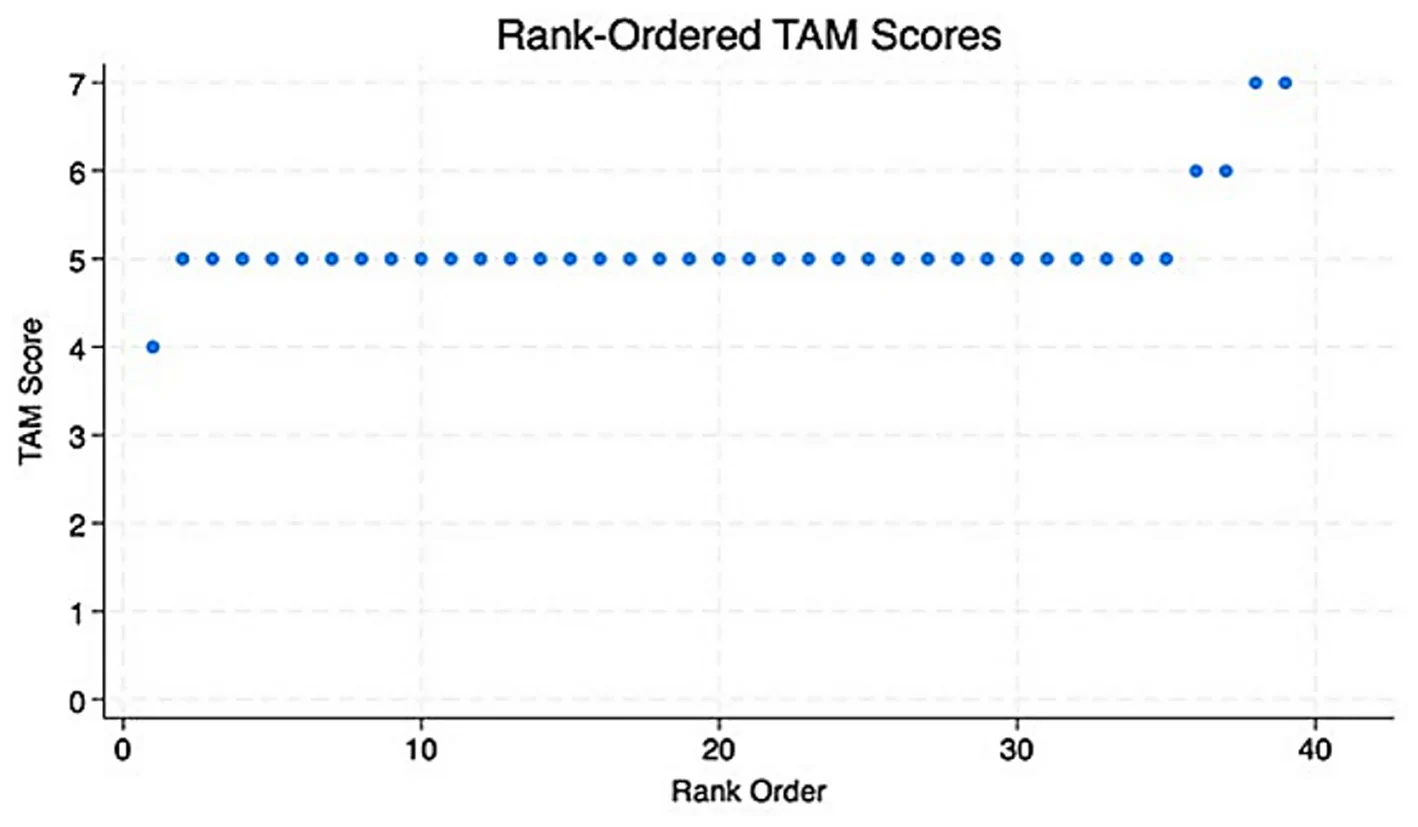
Scatterplot of rank-ordered technology acceptance model (TAM) questionnaires.

The mean TAM score was 5.13, with more than 70% of respondents rating the mobile application software ≥5. (Figure 7). Regression analysis demonstrated that familiarity with digital-payment systems (*p* = 0.0337) and preferred language during Resilience Force communication (*p* = 0.0439) were significant predictors of TAM score, indicating higher acceptance among workers accustomed to using mobile financial tools and those engaging primarily in Spanish-language context.

### 3.4 Predictors of usability and acceptance

The Spanish Language preference for phone operation was significantly associated with SUS (*p* = 0.0287), indicating that linguistic accessibility influenced perceived usability. Familiarity with digital payment methods was significantly associated with higher TAM scores (*p* = 0.0337). No significant associations were observed with age, sex, or education.

## 4 Discussion

Although much work has been done into the incorporation of disaster management into e-technology products (15–17), *Pocket Ark* is unique in that it addresses both the pre-disaster and post disaster needs of the community. Moreover, *Pocket Ark* is focused specifically on the health and safety needs of the skilled workforce that is needed to restore community back to functioning. This study evaluated the usability, technology acceptance, and knowledge improvement associated with *Pocket Ark 2.0* among reconstruction workers engaged in post-disaster recovery (6). Findings demonstrated strong usability (mean SUS 83.9) and high technology acceptance (mean TAM 5.13) within a predominantly Spanish-speaking, Hispanic workforce with moderate digital literacy. Although knowledge gains were modest (mean increase of 0.42 points, *p* = 0.24), the training reinforced core occupational safety principles and wage-protection awareness. These results underscore the effectiveness of a simulation-based, bilingual digital intervention in engaging a vulnerable labor population that remains largely excluded from formal occupational-health training. By combining e-learning modules with geospatial resource mapping and communication tools, *Pocket Ark 2.0* represents a unique model of worker-centered technology aimed at bridging gaps in safety education, preparedness, and advocacy for disaster-response personnel.

The outcomes of this study can be interpreted through the behavioral and organizational frameworks that guided *Pocket Ark 2.0*’s development. Grounded in Social Cognitive Theory, the intervention aimed to strengthen self-efficacy and behavioral capability through interactive, scenario-based learning (7, 8). Reinforcement of appropriate PPE use, wage documentation, and hazard recognition likely contributed to the positive user experience and sustained engagement. Moreover, the finding that those with greater confidence with using digital data had a higher user acceptance of the mobile application software agrees with the initial self-efficacy of the individual being a driver for acceptance of this new technical adaptation of the reconstruction worker’s approach to conducting business operations. From an organizational standpoint, the CFOIA highlights how partnership with an advocacy organization, rather than a formal employer, created an enabling environment for technology adoption (9). This dual-level approach fostered both individual learning and collective reinforcement through peer discussion and social norms, supporting feasibility of community-based digital tools for workforce training in disaster contexts (6).

A key strength of this study was its community-engaged design and bilingual facilitation, which ensured cultural and contextual relevance for participants. Integrating the simulation within the Resilience Force’s ongoing safety-training sessions enhanced ecological validity and improved participation among a difficult-to-reach workforce.

### 4.1 Limitations

There are several limitations to this study. The modest sample size and convenience-sampling approach restricts generalizability, and the single-group post-test design precludes causal inference regarding learning gains. Additionally, the use of identical pre- and post-test items may have introduced testing bias by directing participant attention to key concepts emphasized during the intervention. Course completion time and variability were not systematically measured, limiting assessment of participant engagement and efficiency. Exclusion of nine participants due to incomplete surveys or technical issues may also have introduced selection bias, though descriptive inclusion of these cases mitigated loss of information. Furthermore, the homogeneity of the sample, 100% Hispanic and largely Spanish-speaking, limits application of results to other linguistic or cultural groups. Future studies employing randomized or longitudinal designs could better assess long-term knowledge retention, behavioral change, and real-world safety outcomes.

Despite these limitations, *Pocket Ark 2.0* demonstrates promise as an occupational-health intervention capable of combining education, decision-support, and advocacy within a single mobile platform. The significant influence of language accessibility and prior digital familiarity on usability and acceptance suggests that tailoring mobile-health interventions to users’ linguistic and technological backgrounds is critical for effectiveness (14). Beyond individual training, this approach aligns with current public-health priorities emphasizing scalable, technology-enabled systems for preparedness and workforce protection (18). Future iterations should explore integration of real-time feedback features, long-term knowledge retention, performance analytics, and data dashboards for supervisors and advocates to monitor training uptake and resource needs. Additional research development may also take place regarding use of previously developed app for the general public including additional community resources, volunteer engagement opportunities, and reporting site specific disaster to public health authorities (16). Expanding deployment across multiple geographic regions and incorporating diverse worker populations will further validate generalizability.

*Pocket Ark 2.0* illustrates how mobile technology can operationalize policy guidance from agencies such as OSHA and National Institute for Occupational Safety and Health (NIOSH) by delivering culturally relevant, field-based training directly to workers (4, 5). Wider implementation could support compliance with occupational-safety standards while empowering workers through accessible information on rights, wages, and protective measures. Embedding such tools within community organizations and emergency-response systems may enhance coordination, sustainability, and accountability in disaster recovery efforts. Leveraging digital platforms for training, safety communication, and evaluation reflects an emerging best practice for promoting equity and resilience in the disaster-response workforce (15–17).

## 5 Conclusion

*Flood recovery workers are at risk for numerous occupational hazards. Pocket Ark 2.0*, an e-learning and decision-support application for reconstruction workers, demonstrated measurable knowledge gains, strong usability, and high technology acceptance in this evaluation. Iterative refinements guided by social behavior theory and the CFOIA framework contributed to these outcomes by aligning the mobile application software with both worker-level and organizational-level needs. The findings of this pilot program confirm the feasibility of *Pocket Ark 2.0* as a scalable intervention to enhance safety, preparedness, and advocacy in disaster recovery settings. Continued evaluation in larger and more diverse cohorts will be critical to establishing its long-term impact on occupational and public health preparedness.

## Data Availability

The raw data supporting the conclusions of this article will be made available by the authors, without undue reservation.
